# Introduction to the special issue on oncodermatology

**DOI:** 10.1016/j.jdcr.2025.01.009

**Published:** 2025-03-02

**Authors:** Meghan Heberton

**Affiliations:** Dermatology, The University of Texas Southwestern Medical Center, Dallas, Texas

**Keywords:** medical dermatology, oncodermatology

*To the Editor:* Oncodermatology is a rapidly growing field dedicated to the dermatologic concerns of patients living with cancer. Cancer therapy associated side effects form the backbone of the specialty. Oncodermatologists must be able to correctly diagnose adverse cutaneous events, assess and communicate severity, and participate in critical treatment decisions while considering therapy mechanism of action. Communicating this complex decision making is complicated by fears that stopping or holding treatments will lead to progressive disease. Furthermore, the stress of having unexpected and new conditions while navigating a cancer diagnosis cannot be ignored. Partnering with patients and providers during these challenging parts of their lives is immensely satisfying and impactful.

This issue captures the diagnostic dilemmas, scope of conditions, and treatment considerations that oncodermatologists regularly address. There are representative cases from multiple treatment modalities including cytotoxic, targeted, and immunotherapies. Some cases represent benign conditions with high impact on quality of life like alopecia and others capture medically severe events such as toxic epidermal necrolysis. Combination of targeted and immunotherapies pose a particular dilemma in proper attribution, which is explored in several articles as well. Unique dermatologic interventions are discussed, such as dupilumab for epidermal growth factor receptor inhibitor associated acneiform eruptions, hydroxychloroquine for immunotherapy associated lichen planus, and even maggots for chronic wounds. As new drugs bring unexpected side effects to the clinics and inpatient setting, the field evolves. Examining cases like these can afford familiarity to practitioners new to these drugs and en masse can suggest trends or areas for future research focus. Publishing these experiences and portraying the medical considerations that go into everyday practice is also critical for educating other specialties involved in these cases. There is limitless potential for progress so that we can better serve our patients.

Many thanks to the authors who submitted their work, the patients who allowed their cases to be shared, and to the reviewers who generously donated time and expertise in developing this issue.

## Conflicts of interest

None disclosed.

